# Are You “Gazing” at Me? How Others' Gaze Direction and Facial Expression Influence Gaze Perception and Postural Control

**DOI:** 10.3389/fpsyg.2021.730953

**Published:** 2021-12-23

**Authors:** Angélique Lebert, Laurence Chaby, Amandine Guillin, Samuel Chekroun, Dorine Vergilino-Perez

**Affiliations:** ^1^Vision Action Cognition, Université de Paris, Boulogne-Billancourt, France; ^2^CNRS, Institut des Systèmes Intelligents et de Robotique, Sorbonne Université, Paris, France; ^3^Université de Paris, Boulogne-Billancourt, France

**Keywords:** gaze direction, emotion, approach-avoidance, individual traits, action tendencies

## Abstract

In everyday life, interactions between humans are generally modulated by the value attributed to the situation, which partly relies on the partner's behavior. A pleasant or cooperating partner may trigger an approach behavior in the observer, while an unpleasant or threatening partner may trigger an avoidance behavior. In this context, the correct interpretation of other's intentions is crucial to achieve satisfying social interactions. Social cues such as gaze direction and facial expression are both fundamental and interrelated. Typically, whenever gaze direction and facial expression of others communicate the same intention, it enhances both the interlocutor's gaze direction and the perception of facial expressions (i.e., shared signal hypothesis). For instance, an angry face with a direct gaze is perceived as more intense since it represents a threat to the observer. In this study, we propose to examine how the combination of others' gaze direction (direct or deviated) and emotional facial expressions (i.e., happiness, fear, anger, sadness, disgust, and neutrality) influence the observer's gaze perception and postural control. Gaze perception was indexed by the cone of direct gaze (CoDG) referring to the width over which an observer feels someone's gaze is directed at them. A wider CoDG indicates that the observer perceived the face as looking at them over a wider range of gaze directions. Conversely, a narrower CoDG indicates a decrease in the range of gaze directions perceived as direct. Postural control was examined through the center of pressure displacements reflecting postural stability and approach-avoidance tendencies. We also investigated how both gaze perception and postural control may vary according to participants' personality traits and emotional states (e.g., openness, anxiety, etc.). Our results confirmed that gaze perception is influenced by emotional faces: a wider CoDGs was observed with angry and disgusted faces while a narrower CoDG was observed for fearful faces. Furthermore, facial expressions combined with gaze direction influence participants' postural stability but not approach-avoidance behaviors. Results are discussed in the light of the approach-avoidance model, by considering how some personality traits modulate the relation between emotion and posture.

## 1. Introduction

When people are confronted with threatening social situations such as facing an angry person, they acknowledge a number of cues. Gaze direction is an essential cue as it signals the direction of the threat, in particular when combined with specific facial expressions. For instance, a direct gaze combined with an angry face feels more threatening to the observer than a deviated gaze as the gaze focuses solely on the observer. Conversely, a deviated gaze combined with a fearful face often signals a danger in the environment (Adams et al., 2017). Understanding the source of the threat therefore depends on the interpretation of a unique combination of emotion and gaze direction with some mutual influence. Facial expressions mainly influence the way people perceive gaze directions as directed at them or at the environment (Ewbank et al., 2009) and reciprocally the gaze direction of others helps to identify facial expressions (Adams and Kleck, 2003). These situations trigger a series of hormonal and physiological responses in the observer (Scherer, 2005), preparing their organism to act through action tendencies such as fight (approach), flight (avoidance) or freeze (immobilization) (Adams et al., 2006). Furthermore, action tendencies and perception of gaze direction may also depend on other significant factors such as individuals' personality traits (e.g., anger-trait, anxiety-state, extraversion) or emotional states (e.g., anger-state, anxiety-state). For instance, individuals with a high anxiety-trait level are more likely to interpret the gaze direction from a fearful face as directed at the environment (Hu et al., 2017).

Beyond the threat, accurate recognition of facial expressions is fundamental to correctly interpret intentions and individuals' adjustment of social interactions. Interpreting facial expressions is mostly situation-dependent and is carried out in some specific contexts where partners and observers' characteristics and actions must be acknowledged (Russell, 1997). In addition to emotional facial expressions, gaze directions are also among the most emotionally informative cues in social interactions. These two salient social signals are powerful transmitters of information and are essential social cues integrated by the observer when processing the sender's emotions (Adams and Nelson, 2016). Furthermore, recent findings showed that task demands may impact facial emotional processing, demonstrating that when task-relevant, fearful, and angry expressions capture attention more strongly than happy faces (Mirabella, 2018; Mancini et al., 2020). Conversely, task-irrelevant emotional expressions do not produce any behavioral effect. Direct gaze perception guides the attention toward the face and triggers the activation of specific brain regions recruited to interpret emotions and encode face and eye movements (Wicker et al., 2003; George and Conty, 2008). Conversely, a deviated gaze shifts the observer's visual attention to the gazed-at location. On other executive functions, Marino et al. (2015) demonstrated the influence of gaze direction on inhibition abilities in a social context. Then, the observer needs to correctly understand the emotion and gaze direction of others because it signals their behavioral intentions to approach them or avoid something from the environment (Adams and Kleck, 2003).

According to the shared signal hypothesis proposed by Adams and Kleck (2003), emotion processing is enhanced as the gaze direction matches the motivational orientation of an expressed emotion. Expressions such as happiness or anger are perceived more rapidly and with a higher level of arousal by the observer when combined with a direct gaze because such expressions may be associated to approach motivation (Adams and Kleck, 2003, 2005; Willis et al., 2011; Pönkänen and Hietanen, 2012). Conversely, sad, disgusted, and fearful expressions are perceived more rapidly and with a higher level of arousal when combined with a deviated gaze and may be associated to avoidance motivation (Adams and Kleck, 2003, 2005; Sander et al., 2007). In addition, a neutral face with a direct gaze can be either categorized as anger or happiness while a neutral face with a deviated gaze can be either categorized as fear or sadness (Adams and Kleck, 2005). Nevertheless, Pönkänen and Hietanen (2012) considered neutral expressions as approach-oriented since they are perceived as conveying a higher level of arousal with a direct gaze than when combined with a deviated gaze. These observations highlight the importance of gaze direction in emotion processing.

However, some studies failed to demonstrate some modulation of emotion categorization or arousal by gaze direction. Indeed, the categorization of happy, angry but also sad and fearful faces lead to shorter reaction times when combined with a direct gaze compared to a deviated gaze (Graham and LaBar, 2007; Bindemann et al., 2008). In addition Willis et al. (2011) did not observe any modulation of the arousal by the gaze direction in response to fearful, sad, and disgusted faces. It appears that experimental methods (task type, task relevance, selected database, etc.) account for the observed discrepancies in the results looking at the gaze's effect on emotion processing.

Reciprocally, emotions may also modulate the way one perceives gaze direction. This effect cannot be examined in a binary manner presenting either direct or deviated gaze modalities (Ewbank et al., 2009). Because gaze direction can be ambiguous, discriminating gaze direction may thus be a complex task to complete in everyday life. Due to the multitude of iris positions in the eye, it is necessary to use a finer tool to obtain a more accurate estimation of the gaze direction that is specific to an individual in any given situation. The cone of direct gaze [CoDG, Gamer and Hecht (2007)] refers to a psychophysical index characterizing the extent of gaze deviations that participants may interpret as a direct gaze. The CoDG can be calculated in a gaze categorization task consisting of presenting faces with direct, completely deviated or intermediate (i.e., ambiguous) gaze directions. Participants are then asked to indicate for each gaze direction whether the presented faces are looking to their left, straight ahead or to their right, respectively. The width of the gaze cone is determined from the crossover points between the response proportion for the direct and left gaze direction and the response proportion for direct and right gaze direction. A wide cone corresponds to an increased probability of perceiving a direct gaze, while a narrow cone is associated with an increased probability of perceiving a gaze directed at the environment (Ewbank et al., 2009). Some studies reported a wider CoDG in response to angry faces (i.e., observers perceived faces looking at them over a wider range of gaze directions) compared to fearful and neutral faces (Lobmaier et al., 2008; Ewbank et al., 2009). Lobmaier et al. (2008) found that happy faces are more likely to be interpreted by the observer as looking at them than angry, fearful, or neutral faces. A narrower CoDG is observed in response to fearful faces compared to neutral and angry faces (Jun et al., 2013).

In addition, other factors may modulate the perception of the direct gaze direction. For instance, changes in distance to others may affect gaze direction processing: the further away the individual stands from the face, the more likely they will perceive the gaze as directed at them (Gamer and Hecht, 2007). Furthermore, when situations are uncertain or when visual information is reduced, individuals tend to perceive the gaze as directed at them (Mareschal et al., 2013). Internal factors such as the observer's personality traits and emotional states may also modulate their perception of gaze direction. In participants with high anxiety-trait scores, a wider CoDG was observed in response to angry expressions (compared to fearful and neutral expressions). Also, a narrower CoDG was reported in participants with high anxiety-trait scores in response to fearful faces (compared to individuals with low anxiety-trait scores), thus suggesting hypervigilance toward a potential threat coming from the environment (Hu et al., 2017). A wider CoDG was observed in participants with social phobia viewing neutral faces when compared with healthy participants (Gamer et al., 2011). Additionally, Mathews et al. (2003) showed that attention was guided by the deviated gaze in fearful faces more than in neutral faces, but only in high anxious-trait participants.

Thus, emotion combined with gaze direction constitute essential cues to process the environment since they simultaneously allow observers to evaluate others' intentions and detect external cues that may be favorable or unfavorable or even represent a threat to them. From an evolutionary perspective, these mechanisms are essential and ensure the adoption of adaptive behaviors such as approaching appetitive stimuli and avoiding aversive stimuli, thus promoting the survival of the individuals (Darwin, 1872; Elliot, 2006). As observers decode facial signals in a context of social interaction, different motivational responses are triggered, thus leading observers to opt for specific action tendencies such as approaching affiliative or pro-social situations (e.g., a happy face) or avoiding threatening situations (e.g., an angry or disgusted face) (Gea et al., 2014; Kim et al., 2015). Whenever some emotional stimulus is displayed in an individual's perceptual field, observable slight postural changes are likely to precede the action in real life, or “spring to action” (Elliot, 2006). These variations can be considered as an objective measure of action tendencies and may be collected by using a force platform assessing postural control (Lelard et al., 2014; Lebert et al., 2020). Massion (1994) characterizes postural control as a coordinated adjustment of the different body segments through muscle tone, whose multiple functions include stabilization and body orientation in space. Balance is maintained by keeping the center of gravity in the inner part of the sustentation polygon base (i.e., the area bounded by the feet). The center of gravity is a hypothetical, non-material point, and therefore difficult to study. Postural measurements often substitute the study of the gravity center with the analysis of the standing subject's pressure center displacements. According to Lelard et al. (2019), Center of Pressure displacements on the antero-posterior axis (CoP-Y) allow for the quantification of approach and avoidance behaviors. CoP-Y forward displacements may be associated with an approach tendency, whereas CoP-Y backward displacements may be identified as an avoidance tendency. In addition, CoP displacements allow for the calculation of several parameters that can each quantify postural stability, namely, area, total sway path length and standard deviations of CoP on the medio-lateral (SD-X) and antero-posterior (SD-Y) axes.

There appears to be no consensus today on the results from studies that have investigated the effect of emotional stimuli (i.e., static faces, dynamic faces, scenes) on postural stability and approach-avoidance behaviors. Some studies found reduced body sway (i.e., greater postural stability) indexed by smaller area, length and/or SD-X/SD-Y in participants who were exposed to unpleasant pictures in comparison to neutral and/or pleasant pictures (Azevedo et al., 2005; Facchinetti et al., 2006; D'Attilio et al., 2013). Stins and Beek (2007) replicated this effect but only with participants in unipedal position while Hagenaars et al. (2014) showed a decrease in length in response to unpleasant films, but not to pleasant or neutral films. Finally, the perception of angry faces that resulted in a marked decrease in SD-Y has been interpreted as a freezing behavior (Roelofs et al., 2010). Freezing behavior can be described as a “physiological and somatic preparation for physical movement” first enabling the detection of relevant information, then mobilizing the whole body, and ultimately triggering “fight or flight” behaviors (Elliot, 2006; Lang and Bradley, 2010). D'Attilio et al. (2013) reported increased area and speed in response to pleasant pictures. Comparably, Brandao et al. (2016) found that the length and speed increased in participants who were shown pleasant and unpleasant films or pictures relative to neutral conditions. Regarding the approach-avoidance behavior, previous studies demonstrated that mutilation pictures led to a backward leaning in comparison with neutral and/or pleasant stimuli (Hillman et al., 2004; Lelard et al., 2014). While Eerland et al. (2012) observed an approach behavior in response to pleasant pictures and an avoidance behavior in response to unpleasant picture, Perakakis et al. (2012) identified an avoidance of pleasant, unpleasant and neutral pictures. In addition, Gea et al. (2014) showed an approach behavior in participants exposed to happy and pain dynamic faces. Other studies found little or no emotion effect on the CoP-Y (Azevedo et al., 2005; Stins and Beek, 2007; Lebert et al., 2020).

Finally, postural control was also found to be affected by participant's emotional states, such as anxiety, empathy or fear of falling (Roelofs et al., 2010; Gea et al., 2014; Lelard et al., 2014; Lebert et al., 2020). For instance, Roelofs et al. (2010) found that the decrease in the participants' body sway was correlated with their anxiety level. As the empathy score rose, as Gea et al. (2014) observed in their study, the approach behavior increased in response to happy faces. Lebert et al. (2020) found that high levels of extroversion and neuroticism were associated with an avoidance behavior of fear and anger. As the cognitive empathy and emotional responsiveness score rose, as Lebert et al. (2020) demonstrated that the avoidance behavior increased in response to anger and disgust.

### 1.1. Study Goals

This study aims primarily to gain further knowledge of how the combination of others' gaze direction across a range of gaze directions (direct or deviated) and emotional facial expressions (i.e., happiness, fear, anger, sadness, disgust, and neutrality) impact the observer's gaze perception and postural control. To that end, we set up a protocol allowing for the exploration of emotion effect on gaze perception while considering the participants' individual traits and states using personality questionnaires. Participants were asked to complete (i) a gaze categorization task (ii) a postural passive task during viewing of emotional faces with direct or deviated gaze during which COP displacements were recorded. The gaze categorization task was performed before the postural blocks and then interspersed between each postural block to examine whether the previously seen blocks influenced gaze perception. During the gaze categorization task, participants were asked to categorize the gaze as direct or deviated of each facial expressions across a range of gaze deviations (-9, -6, -3, 0, +3, +6, +9 pixels). We used the CoDG psychophysical index to refer to the width over which an observer feels someone's gaze is directed at them. Examining closely the CoP postural parameters, we investigated the emotions effect combined with a direct or a deviated gaze on postural stability and action tendencies. We also attempted to find out whether the CoDG and the postural control may be modulated by some individuals' traits and states. Our research question may be stated as follows: How can others' emotions (i.e., happiness, fear, anger, sadness, disgust, and neutral) and gaze direction (direct or deviated) affect individuals' gaze perception and postural control on the grounds of personality traits (i.e., anxiety, anger, and Big Five)?

To address this issue, we hypothesized that emotions conveyed by faces may affect gaze perception. In accordance with the shared signal hypothesis (Adams and Kleck, 2003) and previous studies conducted on the categorization of gaze directions (Lobmaier et al., 2008; Ewbank et al., 2009; Jun et al., 2013), we predicted a wider CoDG in response to angry and happy faces, and a narrower CoDG in response to fearful faces. Since disgusted and sad faces may be considered as an avoidance-oriented expression, as Adams and Kleck (2003, 2005) showed, we expected a narrow CoDG in response to these emotions. Furthermore, in some studies, neutral expression may be considered as approach-oriented or avoidance oriented depending on the direction of the gaze displayed by the face (Adams and Kleck, 2005; Willis et al., 2011). However, in Pönkänen and Hietanen (2012) study, neutral expression were associated with a higher level of arousal when combined with a direct gaze than with a deviated gaze, we therefore expected a wide CoDG in response to this expression. We also hypothesized the perception of postural blocks presenting either a direct or a deviated gaze as accentuating the width/narrowness of the cone in accordance with the related motivational tendencies. For instance, the CoDG in response to anger (related to approach) was anticipated to be wider after seeing angry faces with a direct gaze compared to a deviated gaze (Adams and Kleck, 2003).

Based on the shared signal hypothesis (Adams and Kleck, 2003), we assume that postural stability is influenced by the combination of emotional facial expression and gaze direction. We assume that when the combination of the motivational tendency related to emotion and the one related to gaze direction do not match, the perceptual processing would become more complex, affecting posture maintenance and reflected by an increase in postural oscillations (i.e., area of COP displacements). Given that happy, angry, and neutral faces would be better processed when associated with a direct gaze and that sad, disgusted, and fearful faces would be better processed with a deviated gaze, we assumed that these conditions would likely lead to a better postural stability compared to the reversed and therefore incongruent conditions (Adams and Kleck, 2003, 2005; Sander et al., 2007; Willis et al., 2011; Pönkänen and Hietanen, 2012).

Furthermore, we foresaw emotions to have some significant effects on the CoP-Y. In accordance with Gea et al. (2014), we hypothesized approach behavior to be triggered by happy and sad faces relative to other emotions. Considering that results on other emotions in the literature have long been a matter of debate, our hypotheses were essentially exploratory. Also, we assumed that approach and avoidance behaviors could be modulated by individual traits and states. In line with previous studies (Roelofs et al., 2010; Ponari et al., 2013; Lelard et al., 2014; Hu et al., 2017; Lebert et al., 2020), we further conducted exploratory analysis to examine whether the CoDG and postural control parameters may be modulated by some individuals' traits and states.

## 2. Materials and Methods

### 2.1. Participants

Eighty undergraduate participants in total were enrolled in this study. Participants with neurological, psychiatric or postural disorders (scoliosis, recent surgery, etc.) or with a depression score over 17 at the Beck Depression Inventory (Beck et al., 1996) were not included in the study. The significant cut-off regarding the depression score is often used in studies involving emotions owing to the fact that emotional processing can be affected by the presence of significant depressive symptoms (Chaby et al., 2015; Dalili et al., 2015). The data from ten participants were removed because they did not perform the gaze categorization correctly (CoDG not treatable since participant provided the same answers irrespective of the conditions). In addition, five participants with stabilometric parameters values greater than three standard deviations beyond the group average were excluded from the following analysis. We also inspected the individual time courses of the CoP-Y and excluded data from thirteen participants showing some loss of postural stability due to erratic movements (moving their lower limbs, self-touching, etc.). Finally, the data from fifty-two participants (46 females; 6 males) were analyzed (mean age = 20.3 ± 2.7 years old). All participants had normal or corrected-to-normal vision.

The study protocol was approved by the ethics committee from the Université de Paris (reference number n° IRB : 20130500001072). All participants were informed about the procedure prior to the experiment and provided their written informed consent. Participants received course credits for their participation.

### 2.2. Self-Report Measures

The assessment of individual traits and states was carried out using several self-questionnaires. The *STAI-Y* (Spielberger, 1993) consisted of 40 items, half of the items assessed state anxiety while the other half measured trait anxiety. The *STAXI- II* (Spielberger, 1999) was composed of three parts: the first part (15 items) assessed state anger while the second part (10 items) measured trait anger. For the third part, participants completed 32 items by checking the box that best described their usual reactions when they are angry. Finally, the *BIG Five BFI-FR* (Plaisant et al., 2010) consisted of 44 questions assessing the five core human personality traits of neuroticism (avoidance behavior and emotional instability), extraversion (approach tendency and positive emotionality), openness (tolerance and novelty attraction), agreeableness (altruism and high regard for relationships with others), and conscientiousness (inhibition, control, and low impulsivity).

### 2.3. Stimuli

In this study, face stimuli were color virtual expressive Caucasian faces from different genders and levels of arousal. Faces had been generated using the FaceGen Modeller 3.5 software. The hair was removed so that only the central face area was visible. A pre-test was conducted from an independent sample of fifty participants who evaluated 456 Caucasian faces of both young males and females (nineteen identities × four levels of arousal × six emotions). Participants were asked to identify the valence and to rate (using a 10-point Likert scale) the arousal and the natural appearance of the face on a range from 1 (not at all) to 10 (very much). Based on the percentage of correct answers and considering the homogeneity of the different levels of arousal and the degree of naturalness of the face, ten identities were selected (5 women, 5 men), each one expressing six facial expressions (joy, fear, anger, sadness, disgust, and a neutral expression).

Regarding the gaze categorization task, the gaze direction of each face was modified using Facegen. The iris position of both eyes was shifted by 3, 6, or 9 pixels to the left or to the right (similar to Ewbank et al., 2009).

### 2.4. Material

Gaze categorization was performed using a Microsoft SideWinder Plug and Play GamePad (USB) joystick. The antero-posterior (AP) and medio-lateral (ML) displacements of the CoP were recorded using a force platform (AMTI: AccuSway+^®^) and allowed the calculation of the following postural parameters: (i) the surface area of the CoP displacements (in cm^2^), (ii) the mean position of the CoP on the antero-posterior axis (CoP-Y, in cm). The Area provided a measure of postural stability while the mean CoP-Y provided information about the displacement toward, or away from the stimuli and could therefore be considered as an index of the action tendencies. Data were collected at a frequency of 100 Hz. Faces were projected on a Dell screen (1920 × 1200 pixels resolution), placed at a distance of 1 m from every participant and positioned at eye height.

### 2.5. Procedure

**Figure 1:**
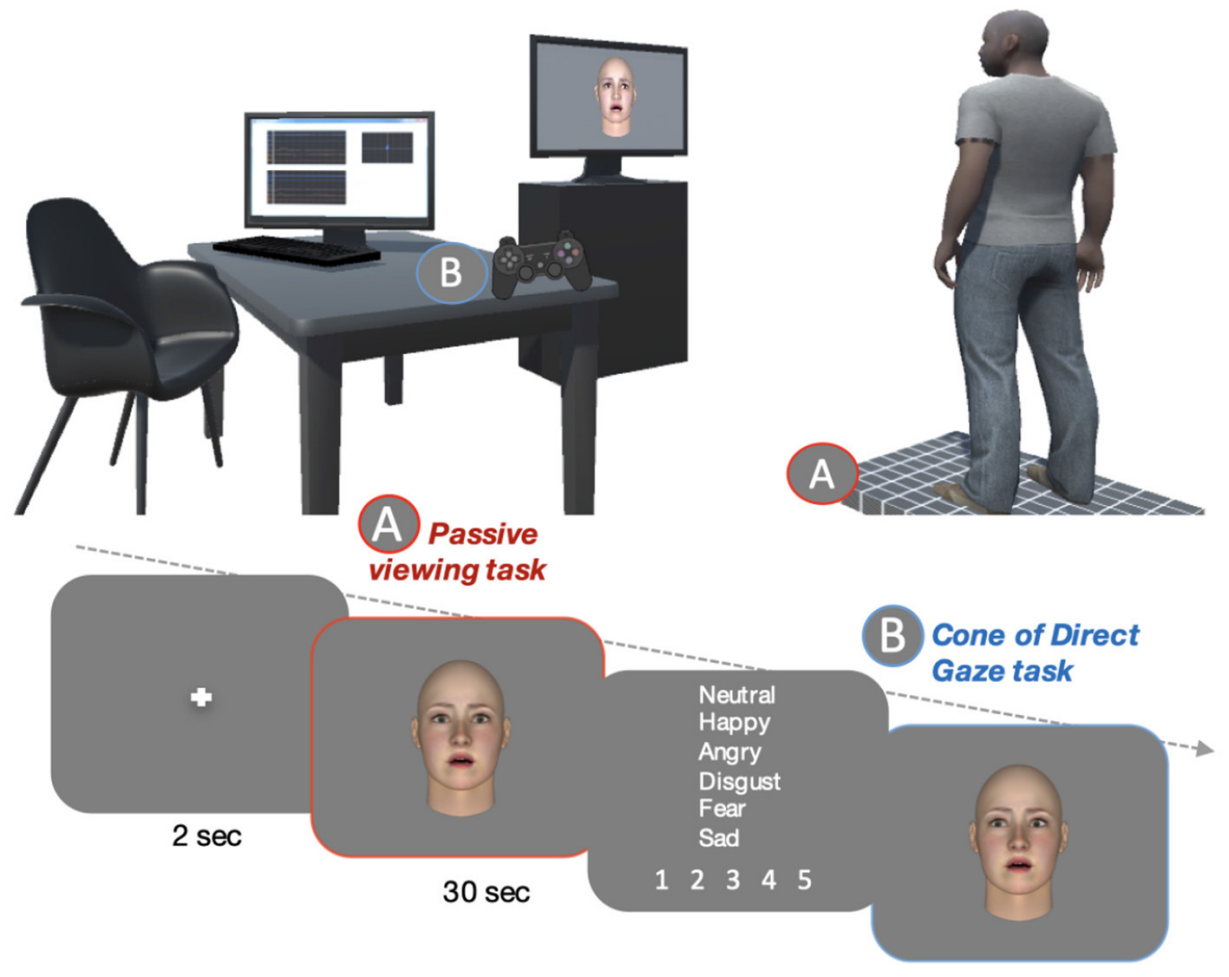
Schematic representation of the experimental tasks: postural passive task during viewing of emotional faces on the force platform **(A)** Gaze categorization using the joystick **(B)**. The dotted arrow indicates the flow of time.

The experimental procedure was performed in a quiet room with a constant luminosity and consisted of two tasks: a gaze direction categorization task (without postural recording) and a postural passive task (with postural recording) during viewing of non-social stimuli (empty screen or fixation cross) or social stimuli (emotional faces with direct or fully deviated gaze) (see Figure 1).

#### 2.5.1. Initial Gaze Categorization

Participants first signed a written consent form and completed health and depression questionnaires. They were then placed on a force platform with their feet hip-width apart and their arms at their sides to maintain a comfortable posture. The position of each foot was marked on the platform to ensure the reproducibility of the posture.

Subsequently, participants performed an initial gaze categorization task (IGCT), during which posture was not recorded. Using the left, middle, and right buttons on the joystick, they indicated whether they considered the face looked to their left, their right, or straight ahead. This task consisted of 504 trials presented randomly: 6 identities (from the 10 identities) repeated twice × 6 emotions × 7 gaze directions. After completing the gaze categorization, participants filled out the questionnaires.

#### 2.5.2. Postural Passive Task During Viewing of an Empty Screen and a Fixation Cross

At this point, participants moved back to the force platform while their postural data were recorded as they performed a postural passive task during viewing of an empty screen and a fixation cross for 30 s each.

#### 2.5.3. Alternating the Postural Passive Task During Viewing of Faces With the Gaze Categorization Task

We created twelve blocks of emotional faces, each including two tasks. Firstly, in the postural passive task, several postural parameters were recorded while participants viewed emotional faces with either a direct gaze or a deviated gaze. Secondly, participants performed gaze categorization, without postural recording. The procedure for each experimental block is detailed below.

To begin with, the postural recording started with the fixation cross appearing on the screen for 2,000 ms. Then the fixation cross was followed by faces from the ten different identities displaying one of the six emotions, with either a direct gaze or a deviated gaze (maximum deviation of 9 pixels allowing little or even no ambiguity as to the deviation of the gaze direction) lasting 30 s. Participants were instructed to simply look at the faces while keeping a natural position with their arms positioned along the body. At the end of the postural recording, participants were instructed to categorize the emotion perceived using the predefined list of the 6 possible expressions, and to rate the arousal of that emotion on a 5-point scale. Participants then performed a similar gaze categorization as in the first step but only with the emotion presented in the preceding postural passive task. Each block consisted of 84 trials including one of the six emotions displayed with the seven gaze directions and was presented twelve times.

All stimuli were presented in a randomized order to each participant. The order of presentation for each block was counterbalanced across subjects with identical conditions maintained in no more than two consecutive trials. The task was programmed and implemented using Opensesame (Mathôt et al., 2012).

### 2.6. Statistical Analysis

**Figure 2:**
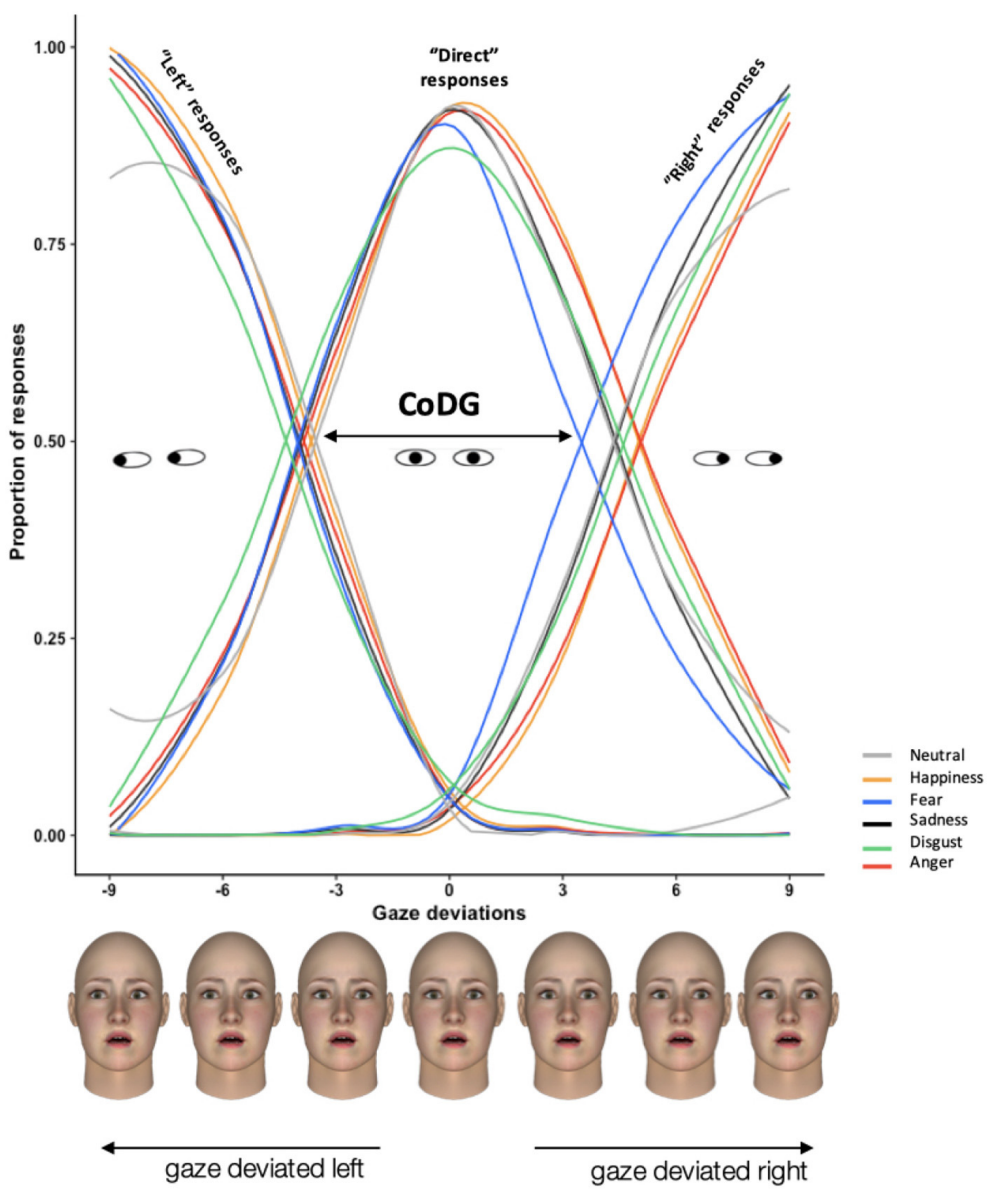
Plot showing mean fitted left, direct and right responses as a function of gaze direction for the six emotions across all participants. The arrow represents the width of cone. Each degree of gaze deviation is illustrated with a corresponding example face.

Primary statistical analyses entailed exploring CoDG width. The calculation of the CoDG allowed for the determination of three logistic functions that provided information on the probabilities for an individual to categorize a gaze as either direct, left or right at any given moment (see Figure 2). Based on this calculation, we defined two thresholds corresponding to the crossover points between the fitted direct and right functions and the crossover point between the fitted direct and left functions. These thresholds delimited the cone and thus determined its width. As the laws of psychophysics have demonstrated, a widening of the direct gaze cone corresponds to increased probabilities for perceiving a direct gaze, whereas the narrowing of this cone is linked to increased probabilities for perceiving a deviated gaze (Ewbank et al., 2009).

Subsequently, we conducted a repeated measures analysis of variance (ANOVA) on the CoDG width using 3 (Gaze categorization steps: initial gaze categorization, gaze categorization after viewing direct gaze - deviated gaze) × 6 (Emotions: happy, fear, anger, sadness, disgust, and neutral expression) conditions. Planned comparisons were used for paired comparisons and Spearman correlation coefficients between CoDG width and scores related to individual traits were also computed.

Secondary analyses entailed exploring the following postural parameters variations: the Area indexing the postural stability, the CoP-Y reflecting action tendencies behaviors. These postural parameters were analyzed using 2 (Gaze directions: direct gaze, deviated gaze) × 6 (Emotions: happy, fear, anger, sadness, disgust and neutral expression) repeated measures analysis of variance (ANOVA). Planned comparisons were used for paired comparisons. Furthermore, correlation coefficients between postural parameters and individual traits were computed.

All the analyses were performed using the R-statistical environment software (R Core Team, 2013). ANOVAs were computed using the “afex” package (Singmann et al., 2015), then planned comparisons were performed with the “emmeans” package (Lenth, 2019). To provide clarity, the corrected degrees of freedom were reported, with the *p*-value aligned with the Huynh-Feldt adjustment. Spearman correlations were computed to further explore the relationship between individual traits and CoDG width or postural parameters. Since we had specific hypotheses on the ANOVAs and then on the planned comparisons we corrected our *p*-values using the Bonferroni corrections. However, given that our correlations were exploratory, we did not correct them (Bender and Lange, 2001). We computed the eta-squared (η^2^) for each planned comparison. A significance level of *p* = 0.05 was used for all statistical analyses.

### 2.7. Results

#### 2.7.1. CoDG

**Figure 3:**
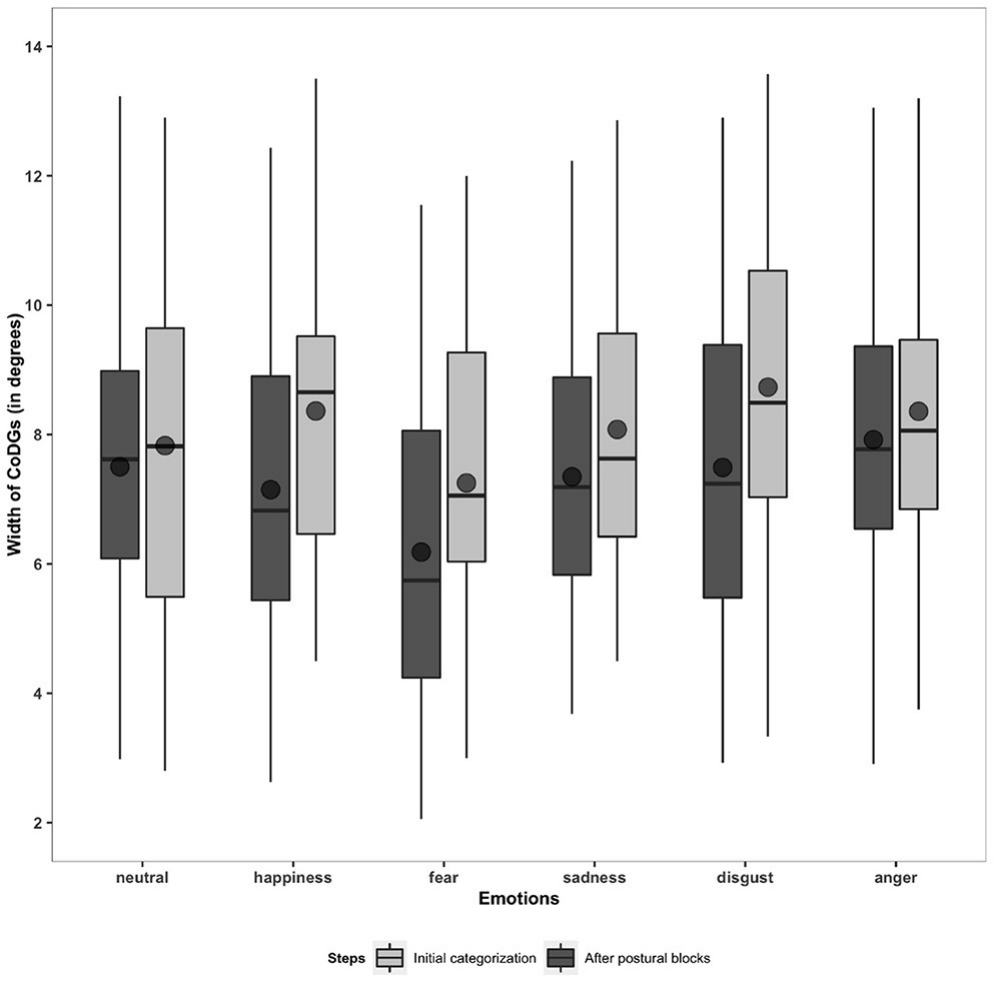
Boxplots and means for CoDGs width (in degrees) based on steps (initial categorization, after postural blocks) and emotions (neutral, happiness, fear, sadness, disgust, and anger).

First, we examined whether the CoDG width was influenced by the Emotions and the Gaze categorization steps. The ANOVA revealed a main effect of the Emotions [*F*_(4.57,233.13)_ = 16.61, *p* < 0.001], a main effect of the Gaze categorization steps [*F*_(1.70,86.62)_ = 11.35, *p* < 0.001], and a Gaze categorization steps × Emotions interaction [*F*_(8.13,414.39)_ = 3.03, *p* = 0.007]. Interestingly (see Figure 3), the CoDG in response to fearful faces (M = 6.72, SD = 2.16) was significantly narrower than the CoDG with all the other emotions (M = 7.88, SD = 1.95, *p* = 0.001, η^2^ = 0.5). The angry (M = 8.14, SD = 2.10) and disgust (M = 8.11, SD = 2.50) CoDGs did not differ from each other (*p* >0.05) but were significantly wider (M = 8.12, SD = 2.17) in regard to neutral, happy, and sad CoDGs (M = 7.71, SD = 1.90, *p* = 0.01, η^2^ = 0.5).

Planned comparisons revealed that the CoDG was wider in the initial gaze categorization (M = 8.10 SD = 2.13) than after viewing direct/deviated gaze in postural blocks (M = 7.26, SD = 2.08, *p* = 0.001, η^2^ = 0.4). This interaction showed that this difference was only significant for happy (IGCT: M = 8.36, SD = 2.33; after viewing direct/deviated gaze: M = 7.15, SD = 2.29, *p* = 0.001, η^2^ = 0.2), fearful (IGCT: M = 7.25, SD = 2.31; after viewing direct/deviated gaze: M = 6.18, SD = 2.52, *p* = 0.002, η^2^ = 0.2), and disgusted (IGCT: M = 8.73, SD = 2.86; after viewing direct/deviated gaze: M = 7.49, SD = 2.53, *p* < 0.001, η^2^ = 0.2) faces.

We further explored whether individual variables could modulate CoDG width. To achieve this, we examined correlations between CoDG width with scores obtained from the different self-report measures. Interestingly, the CoDG width appeared to be related to depressive symptoms and anxiety trait scores. As participants displayed fewer depressive symptoms (BDI-II), the CoDG became wider in response to angry (*r* = −0.28, *p* = 0.04) and sad faces (*r* = −0.31, *p* = 0.02) (all steps averaged together), and to disgust (*r* = −0.28, *p* = 0.04) and neutral (*r* = −0.29, *p* = 0.03) faces after the viewing of a deviated gaze in postural blocks. In addition, as the anxiety trait score rose, the narrowing of the CoDG increased in response to sad faces after the viewing of a deviated gaze postural blocks (*r* = −0.42, *p* = 0.001).

#### 2.7.2. Postural Parameters

As participants were liable to move on both the ML and AP axes during the presentation of the initial fixation cross, the postural data were baseline-corrected. All trials started from the same coordinates (0.0) at the beginning of the emotional stimuli presentation. It should be noted that the order of blocks presentation did not have any effect on postural parameters nor did they interact significantly with the gaze directions or the emotions for any of the analyzed postural parameters.

First, we compared the Area in response to the three types of stimuli (empty screen, fixation cross and social stimuli by averaging the six emotions × two directions) to check whether the presence of a more complex stimulation on the screen would lead to a poorer postural stability. The ANOVA performed on the Area showed a main effect of the stimulus type [*F*_(1.85,94.47)_ = 15.13, *p* < 0.001]. Planned comparisons revealed that the Area significantly increased in response to social stimuli (M = 2.07, SD = 1.51) relative to an empty screen and a fixation cross averaged together (M = 1.22, SD = 0.83, *p* < 0.001, η^2^ = 0.5).

**Figure 4:**
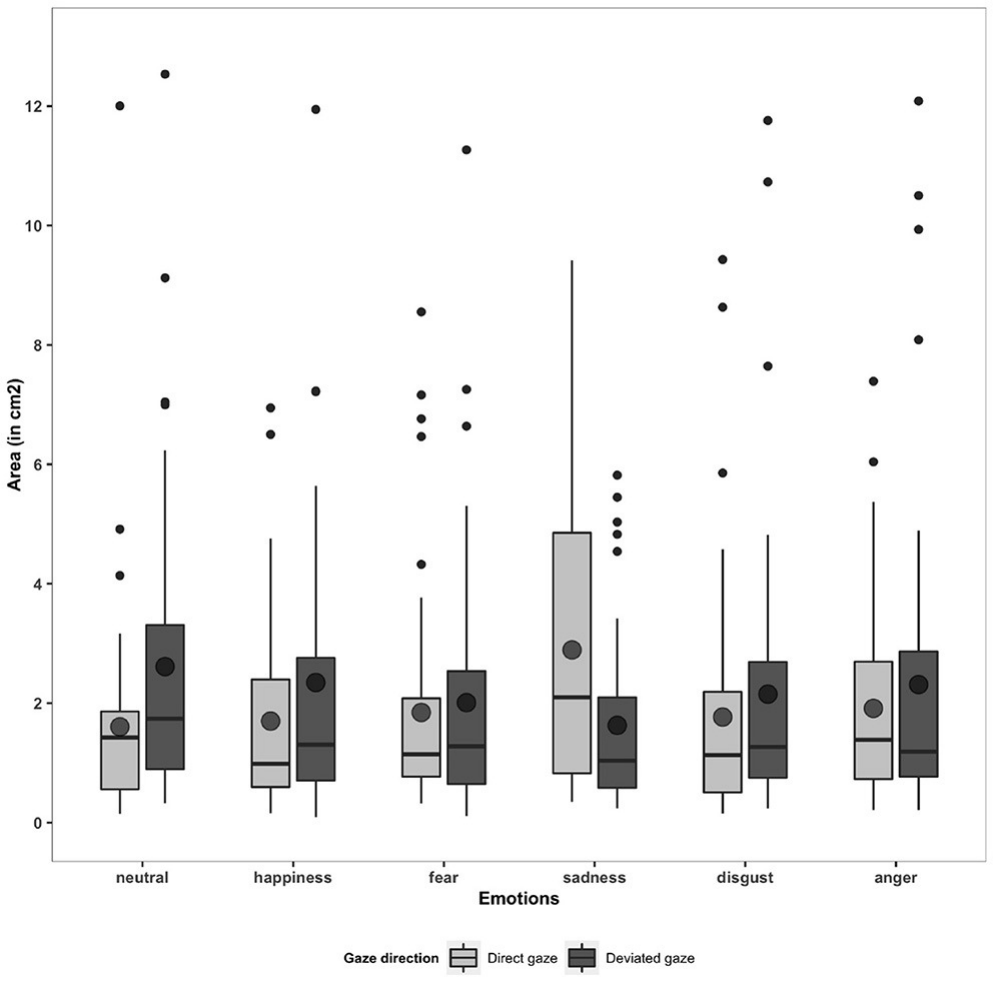
Boxplots and means for Area (in cm^2^) in response to six emotions (neutral, happiness, fear, sadness, disgust, and anger) and two gaze directions (direct and deviated).

Secondly, we examined whether the overall postural stability (Area) and the approach-avoidance tendencies (indexed by the CoP-Y) were influenced by the gaze direction and the emotions. The 2 (Gaze direction: direct and deviated) × 6 (Emotions: happy, fear, anger, sadness, disgust and neutral expression) ANOVA performed on the Area did not show any Gaze direction effect [*F*_(1, 51)_ = 3.21, *p* >0.05] nor any Emotions effect [*F*_(4.41, 225.16)_ = 0.69, *p* >0.05]. However, we observed some significant interaction between Gaze direction and Emotions [*F*_(4.71, 240.28)_ = 6.10, *p* < 0.001] (see Figure 4). Planned comparisons showed an increase in Area in response to neutral faces with a deviated gaze (M = 2.61, SD = 2.51) relative to a direct gaze (M = 1.61, SD = 1.77, *p* = 0.009, η^2^ = 0.2). Conversely, the Area increased in response to sad faces with a direct gaze (M = 2.89, SD = 2.43) relative to a deviated gaze (M = 1.63, SD = 1.41, *p* < 0.001, η^2^ = 0.2).

We further examined whether individual traits might modulate the postural stability of participants. A high score of agreeableness (BFI) was associated with an increase in the Area in response to happy (*r* = 0.29, *p* = 0.03) and angry (*r* = 0.30, *p* = 0.03) faces with a direct gaze. As the STAXI-ECI score (indexing of the anger expression toward ourselves) rose, the Area decreased in response to disgusted (*r* = −0.33, *p* = 0.01) and sad faces (*r* = −0.28, *p* = 0.04) with a direct gaze and neutral faces with a direct (*r* = −0.36, *p* = 0.03) and a deviated gaze (*r* = −0.29, *p* = 0.03).

**Figure 5:**
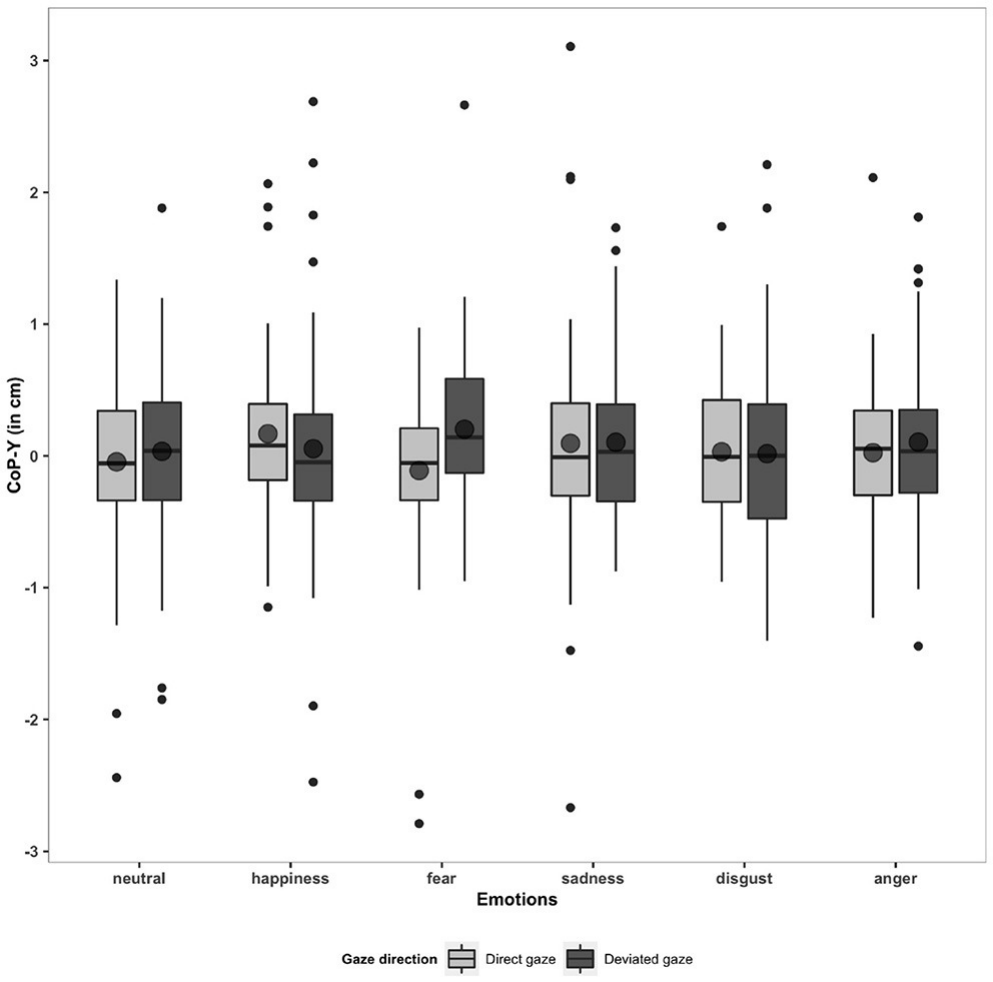
Boxplots and means for CoP-Y (in cm) in response to six emotions (neutral, happiness, fear, sadness, disgust, and anger) and two gaze directions (direct and deviated).

Finally, we did not observe any Emotions [*F*_(4.14, 261.09)_ = 1.78, *p* >0.05] or Gaze direction [*F*_(4.14, 261.09)_ = 1.78, *p* >0.05] effect nor any Faces movements × Emotions interaction [*F*_(4.54, 285.91)_ = 1.73, *p* >0.05] on the CoP-Y (see Figure 5). Interestingly, some correlations between CoP-Y and individuals traits emerged. A high score of Openness (BFI) was associated with an approach behavior in response to happy faces with a deviated gaze (*r* = 0.45, *p* < 0.001). Conversely, a high score of Agreeableness was associated with an avoidance behavior in response to happy faces with a deviated gaze (*r* = −0.29, *p* = 0.04). As the Neuroticism score rose, participants avoided fearful faces with a direct gaze (*r* = −0.42, *p* = 0.001). A high score of anger trait was associated with an approach behavior in response to happy faces with a direct gaze (*r* = 0.29, *p* = 0.03). Furthermore, a high score of anxiety state was associated with an approach behavior in response to neutral faces with a deviated gaze (*r* = 0.28, *p* = 0.04).

### 2.8. Discussion

Successful social interactions are partly based on the combinatory process of relevant emotional cues of others such as emotional faces and gaze direction. Emotional expressions and gaze direction reflect the behavioral intentions of others and trigger differentiated motivational orientations in the observer such as approach-avoidance action tendencies. To our knowledge, no study to date has examined the perception of gaze direction with such a large panel of emotions while considering the influence of individual variables. Also, no author has studied the influence of emotional faces - exhibiting a direct or a completely deviated gaze - on postural control by considering changes in body center of pressure (CoP) displacements.

This study aims primarily to better understand how the gaze direction of others in combination with emotional facial expressions may impact the observer's gaze perception and postural control. A central point in our study is to explore possible modulations between emotions and gaze perception while considering stable personality traits and emotional states (i.e., anxiety, anger, and Big Five). To this end, the range of gaze deviations participants interpreted as looking directly at them was measured using a psychophysics index (i.e., CoDG) in response to six facial expressions (happy, fearful, angry, disgusted, sad, and neutral faces) during an initial gaze categorization task, and after viewing faces with either a direct or a deviated gaze. In addition, participants' postural oscillations in response to emotional faces with a direct or a deviated gaze were measured using a force platform in order to index postural stability and approach-avoidance behaviors. Specific self-reports were used to assess participants' personality traits and emotional states.

Our first finding was that the gaze direction perceived by participants was influenced by the displayed emotion. As anticipated, our study confirms Ewbank et al. (2009) and Jun et al. (2013) findings: wider CoDGs were observed after the viewing of angry and disgusted faces while a narrower CoDG was observed with fearful faces. Both anger and fear expressions signal a threat but from different sources, coming respectively from the sender and from the environment. A direct gaze associated with an angry face is more threatening to the observer than a deviated gaze, as the direction designates the observer as the object of the threat (Adams and Franklin, 2009). Conversely, a deviated gaze associated with a fearful face may signal a danger in the environment, potentially threatening to the observer (Adams et al., 2017). The perception of anger and fear thus led to the establishment of the narrowest and widest cones, respectively, corroborating Mirabella (2018) and Mancini et al. (2020)'s studies which pointed out that fearful and angry expressions strongly capture attention, when task relevant. While we expected disgusted faces to signal an avoidance orientation and lead to a narrower CoDG (Adams and Kleck, 2005), we observed a similar CoDG width in response to disgusted faces and angry faces. These unexpected results may be due to the fact that behavioral reactions to disgusted faces have been reported to be rather complex. Disgust might trigger approach or avoidance behaviors depending on the sources: danger of food consumption, social rejection, or socio-moral violations situations (Seidel et al., 2010; Willis et al., 2011). Furthermore, neutral, happy, and sad CoDGs were wider than a fearful CoDG but narrower than angry and disgusted CoDGs. These findings reinforce the notion that happy and neutral faces may be considered as approach-oriented emotions (Willis et al., 2011) and support the view that fearful and sad faces may be considered as pro-social intentions. These observations support the shared signal hypothesis Adams and Kleck (2003), highlighting the joint effect of emotion and gaze deviation on gaze direction categorization. The combination of the motivational orientation of emotional facial expression and gaze direction would influence the mechanisms underlying perceptual processing.

In addition, we observed an effect of gaze categorization steps on the CoDG width. However, this factor interacts significantly with the emotions conveyed by faces. More specifically, the CoDG was wider during the IGCT than after the previous postural passive task during viewing of emotional faces with a direct or a deviated gaze, but only significant in response to happy, fearful, and disgusted faces. As discriminating gaze direction is a complex task to achieve in everyday life, especially when the gaze is ambiguous, we assumed that participants in the initial trials would interpret the facial gaze as being more directed at them than in subsequent trials. Furthermore, the CoDG width did not generally change after the viewing of a direct gaze in postural blocks or a deviated gaze in postural blocks. Overall, the pattern of width CoDG after the viewing of a deviated gaze is similar to the pattern of width for the initial categorization task.

Interestingly, the width of CoDGs was moderately modulated by personality traits and emotional states. Since our correlations are exploratory, results should be taken with caution, nevertheless our observations are consistent with the existing literature on the influence of personality traits (Adams and Kleck, 2005; Spielberger, 2010; Radke et al., 2014). A low score on the depression scale predicted wider CoDGs in response to angry and sad faces (irrespective of the gaze categorization step) and in response to disgusted and neutral faces (after viewing a deviated gaze in postural blocks). In other words, participants with higher depressive tendencies scores (but below the pathological threshold) interpreted gaze directions as a gaze toward the environment in response to neutral, sad, disgusted, and angry faces. Although we did not include participants with high depression score, this observation is consistent with Radke et al. (2014)'s observation revealing some significant association between depressive symptoms and difficulties in behavioral adjustment in response to emotional faces. Finally, a narrower CoDG in response to sad faces after the viewing of a deviated gaze in postural blocks was measured in participants with high anxiety-trait scores. According to Adams and Kleck (2005), perception of sad faces with a deviated gaze is enhanced because it indicates social withdrawal and dejection. Individuals with a high score of anxiety-trait show feelings of apprehension, tension and nervousness (Spielberger, 2010). As a result, such individuals could be biased in categorizing the gaze from sad faces as a deviated gaze, even more so after viewing sad faces with a deviated gaze (i.e., social rejection situation).

While other's emotion and gaze direction modulate the observers' perception indexed by the gaze categorization task, they also impact the observers' motor adjustments as shown by postural measurements. First, an effect of the stimulus type was characterized by postural instability (indexed by the surface area of the COP displacements) in response to social stimuli found to be higher than in response to the fixation cross or in response to the empty screen. These results are consistent with studies that established a link between balance and cognitive processing (Förster and Stepper, 2000; Fraizer and Mitra, 2008; Lacour et al., 2008), as the load of perceptual information presented to participants predicts their instability. According to the non-linear U-shaped interaction model, postural task performance is improved for a simple cognitive subtask, but deteriorated for a more complex cognitive subtask (Brown et al., 1999; Wulf and Prinz, 2001). Since processing a social stimulus is a more complex cognitive task than perceiving an empty screen or a fixation cross, the increase in instability observed corroborates the literature.

Based on the shared signal hypothesis and given our results regarding the influence of the combination of emotion and gaze direction on the perceptual processing reflected by different cone widths, we assumed that this effect would extend to the motor correlates. We expected the surface area of the COP displacements to be modulated by emotions according to the gaze direction. We observed poorer postural stability (i.e., increase in area) in response to neutral faces with a deviated gaze (compared with a direct gaze) and in response to sad faces with a direct gaze (compared with a deviated gaze). According to some previous studies (Adams and Kleck, 2005; Pönkänen and Hietanen, 2012), neutral expression is more accurately perceived in combination with a direct gaze, whereas sadness is more easily identified in combination with a deviated gaze. These authors emphasize the facilitating effect of a direct gaze on the perception of neutral and happy faces, as well as the associated approach motivation, which may also account for the observations on the neutral emotion in our study. Poorer stability associated with these emotions reveal the complexities of perceptual processing due to the incongruence of facial expression with gaze direction. While the shared signal hypothesis implies that the perception of an emotion is enhanced when gaze direction matches the underlying behavioral intent communicated by the expression (Adams and Kleck, 2005), we show here that the emotion in conjunction with gaze direction also plays a role in the motor correlates as indexed by postural stability. However, this effect is more moderate than on the perceptual aspects, and would perhaps involve other mechanisms than those evoked by the shared signal hypothesis. Postural control allows us to observe reactions that are different from those observed in the context of perceptual processing of emotions, and in particular the phenomenon of freezing (i.e., immobilization). The absence of effects in response to angry and fearful faces - two emotions tightly linked to a sense of danger - might be due to the phenomenon of freezing (Adams et al., 2006). Indeed, emotions from angry and fearful faces are more likely to be processed irrespectively of the gaze direction, unlike other emotions, due to their adaptive function. These observations highlight the limitations of the shared signal hypothesis in the context of postural control.

The surface area of the COP displacements, as it appeared in our study, is also moderately influenced by certain personality traits and emotional states. A high score of agreeableness was associated with an increase in the Area in response to happy and angry faces with a direct gaze. As agreeableness is associated with sympathy and compassion (DeYoung et al., 2007), this increase in postural instability might indicate some sensitivity to a happy face looking directly at the observer, usually signaling an invitation to interact socially. This agreeableness score might also reveal some sensitivity to an angry individual with a direct gaze (perceived as a threat). As the STAXI-ECI score (i.e., the indexing of the anger expression toward ourselves) rose, the Area decreased in response to sad and disgusted faces with a direct gaze and in response to neutral faces with a direct or a deviated gaze. The ECI scale assesses how often the experienced anger is suppressed (Forgays et al., 1997). Participants who suppress their angry feelings would be more stable - or even freeze - in response to disgusted faces with a direct gaze, perceived as a direct threat to them. Interestingly, participants provided a similar response to sad faces with a direct gaze, indicating a high degree of vulnerability suggesting a need for help. Furthermore, neutral expressions may conceal some negative meaning (Tronick et al., 1998). As a result, the decrease in the participants' Area may be due to the repression of their angry feelings and desire for confrontation.

Lastly, we did not identify any effects of emotions and gaze directions on the mean CoP-Y position. These results are consistent with the findings of previous studies showing little or no effect of emotions on CoP-Y (Azevedo et al., 2005; Stins and Beek, 2007; Horslen and Carpenter, 2011). Stins and Beek (2007), for instance, observed some modest forward shift of the CoP-Y in response to neutral and unpleasant pictures and no effect for pleasant pictures. Azevedo et al. (2005) and Horslen and Carpenter (2011) did not observe any emotion effect on the CoP-Y in response to pleasant or unpleasant stimuli. One limitation of this study is that our sample is predominantly female, and this imbalance should be considered in our results as some studies have found gender differences in action tendencies, such as a greater avoidance behavior in women than in men in response to unpleasant stimuli (Hillman et al., 2004; Perakakis et al., 2012). However, it should be noted that the literature on the influence of emotional stimuli on posture includes a significant number of studies conducted only in women or with a majority of women (Roelofs et al., 2010; Stins et al., 2011; Eerland et al., 2012; Hagenaars et al., 2012, 2014; Gea et al., 2014).

However, although Lebert et al. (2020) did not report any effect of emotional faces on the CoP-Y, they observed some correlations with individual traits. For instance, high extraversion and neuroticism scores were associated with an avoidance behavior of angry and fear conditions. Gea et al. (2014) reported an approach of happy and pain dynamic faces but also some positive correlation between “empathic concern” and increased amplitudes of forward body movements for happy faces or increased body sway movements for pain faces. Similarly, we observed that approach-avoidance tendencies were shaped by personality traits and emotional states. High scores of extraversion and openness refer to enjoyment of new experiences and to greater tolerance and were associated with an approach behavior in response to happy faces (McCrae and Costa, 1987; Rammstedt and John, 2007; Hughes et al., 2020). Conversely, neuroticism refers to being anxious and to avoidance or flight behaviors and is a good predictor of responding more strongly to negative events (Smits and Boeck, 2006; Schindler and Querengasser, 2019). Thus, the avoidance tendency observed in response to fearful faces with a direct gaze for participants with high neuroticism score is consistent with the literature. We also observed that participants with a high anger-trait score tend to approach happy faces with a direct gaze. As high anger-trait individuals are more prone to state anger and display high levels of approach motivation (Veenstra et al., 2017) and as happy expression and a direct gaze both indicate an approach-orientation, it may be assumed that participants are more likely to adopt an approaching behavior in this condition. Lastly, high anxiety-state scores were associated with an approach of neutral faces with a deviated gaze. This result is surprising given the existing literature and this behavior seems difficult to explain. Li et al. (2019) observed that anxious would pay more attention to emotional faces than non-anxious individuals. Furthermore, Adams and Kleck (2005) showed that a neutral face combined with a deviated gaze can be categorized as fear expression. It is possible in our study that anxious individuals would have inferred the presence of a threat in the environment by interpreting the neutral face as an expression of fear and would therefore have sought to approach others.

As a result, the correlations established between personality traits and approach-avoidance tendencies suggest a great diversity of behaviors in response to the same stimulus, which may account for the lack of significant effect on the average displacement of the center of pressure on the Y axis.

## 3. Conclusion

Overall, this study expands our understanding of how emotional cues such as facial expression and the gaze direction of others modulate the gaze perception and postural control of participants. This research also shows how individual variables influence this process. To our knowledge, this is the first study to examine how emotional faces, including a large panel of emotions, and gaze deviation influence postural control and the perceived gaze direction of others. Furthermore, the originality of this study lies in the investigation of the crosslinks between perception and action that are moderaterely modulated by individual traits and states. Our results corroborate the idea that social cues such as emotional faces or gaze direction are powerful vectors of information and drives for motivating social interactions. Using a psychophysics index (i.e., CoDG), we demonstrated that threatening facial emotions, such as anger and disgust, increased the participants' feelings of being looked at, more than other emotions. Conversely, the perception of the gaze displayed by fearful faces, suggesting a threat, was interpreted as a deviated gaze. Moreover, some individual variables also played a role in the perception of the gaze direction: a low score on the depression scale predicted wider CoDGs in response to angry and sad faces. We also observed some specific adjustments of postural behaviors based on the displayed emotional stimuli. Participants were more unstable in response to incongruent conditions such as the combination of neutral faces with a deviated gaze (although these are approach-oriented emotions) and sad faces with a direct gaze (although this is an avoidance-oriented emotion). Therefore, the influence of emotion and gaze direction on overall postural stability varies according to the motivational orientation associated with these two social cues. In addition, some individual variables such as anger or agreeableness intensities influenced the postural parameters used to quantify postural stability. However, action tendencies reflected by CoP-Y displacements were not modulated by emotion or gaze direction. Interestingly, personality traits and emotional states played a role in approach and avoidance behaviors. High scores of extroversion and openness were associated with an approach behavior in response to happy faces with a deviated gaze while participants with a high anger-trait score tended to approach toward happy faces with a direct gaze. These various observations suggest that perceptual processing and motor correlates are differentiated according to the combination of emotion and gaze direction. The link between emotion and action is complex and needs to be considered through an integrative approach that acknowledges the interaction between several individual factors.

## Data Availability Statement

The datasets presented in this article are not readily available because the data is not available as participants only gave ethical consent for this project, and not for further distribution outside the research team. Requests to access the datasets should be directed to angelique.lebert1@gmail.com.

## Ethics Statement

The studies involving human participants were reviewed and approved by the Ethics Committee of the Universite de Paris (reference number n° IRB: 20130500001072). The patients/participants provided their written informed consent to participate in this study.

## Author Contributions

AL, LC, and DV-P involved in the conceptualization and experimental design for this project. AL, AG, and SC programmed the experiments, created the stimuli, ran the participants, processed the data, and analyzed the data. AL wrote the initial draft of the manuscript with revision from LC and DV-P. All authors contributed to the article and approved the submitted version.

## Funding

This work has been supported by the French National Agency (ANR) (In-PACT, project ANR-19-CE28-0011).

## Conflict of Interest

The authors declare that the research was conducted in the absence of any commercial or financial relationships that could be construed as a potential conflict of interest.

## Publisher's Note

All claims expressed in this article are solely those of the authors and do not necessarily represent those of their affiliated organizations, or those of the publisher, the editors and the reviewers. Any product that may be evaluated in this article, or claim that may be made by its manufacturer, is not guaranteed or endorsed by the publisher.
